# Modeling the functionalized genistein-hyoscyamine derivatives

**DOI:** 10.1038/s41598-025-00371-1

**Published:** 2025-05-13

**Authors:** Rana Abd-ElSalam, Nada A. Khaled, Medhat A. Ibrahim

**Affiliations:** 1 Food and Dairy Science Department, Mansoura University, El Gomhouria St., El Mansoura, 35516 Dakahlia Egypt; 2 Therapeutical Chemistry Department, National Research Centre, 33 El-Bohouth St., Dokki, Giza, 12622 Egypt; 3 Spectroscopy Department, National Research Centre, 33 El-Bohouth St., Dokki, Giza, 12622 Egypt; 4 Molecular Modeling and Spectroscopy Laboratory, Centre of Excellence for Advanced Science, National Research Centre, 33 El-Bohouth St., Dokki, Giza, 12622 Egypt

**Keywords:** Nocturnal enuresis, Genistein, Hyoscyamine, MESP, QTAIM, DFT descriptors and ADME parameters., Materials science, Physics

## Abstract

Due to the significant rise in demand for functional foods and health-conscious alternatives, natural extracts present a promising avenue for exploration and application within the food processing sector. For many individuals suffering from monosymptomatic nocturnal enuresis (MNE) and its accompanying complications, supplying the market with a functional food that aids accelerating resolving this problem will be a very valuable addition. Thus, in this study we aimed to validate and functionalize modeled composite of Genistein-Hyoscyamine as to further employ the best match in food processing sector as a novel food-additive for functional-foods serving enuretic patients. In attempts to model the most chemically favorable and experimentally achievable composite structures we thoroughly studied the parent molecules employing various DFT descriptors, selected electronic and thermodynamic parameters that help foresee and assess the structures’ behavior and stability in various conditions that are common during food processing. Afterwards, composites were primarily assessed through selected ADME parameters in regard to their suitability for ingestion, water solubility, GI absorption, bioavailability score, and synthetic accessibility. Based on the screening of modeled structures, composites number 02 and 04 were found to possess the most favorable structures and characteristics where composite number 02 has shown relatively higher band gap energy and dipole moment as well as slightly more heat capacity; while composite number 04 has shown higher lipophilicity as well as lower TPSA value, less enthalpy, free energy, and entropy, suggesting more stability and bioavailability. Highlighting their suitability for being introduced to food matrices as food-additives in the form of composite bioactive materials.

## Introduction

Genistein is a naturally occurring isoflavone found in soybeans and other members of the *Fabaceae* family and it was first extracted and defined in 1899 out of the *Genista* species to which its name is attributed and it has been utilized via pharmacological synthesis in the nineties to meet the requirements for its use as a nutraceutical^1^.

Naturally, its highest concentrations occur in soybeans. It has been recently studied for being a bioactive natural extract possessing many potential benefits one of which is its reported in vivo effects of increasing vasopressin (antidiuretic hormone) content while consumed orally^2^. Other reported benefits were neuro protective effects, cardiovascular protective effects, as well as being an immunomodulatory, anti-cancer, anti-obesity, anti-diabetic, and anti-osteoporosis agent. Moreover, genistein as a known phytoestrogen has been utilized as a dietary supplement aiding women through physiological changes accompanied to menopause, reported to alleviate incidence of vasomotor symptoms and stress responses^3^. Nevertheless, many recent in vitro studies have highlighted the role of genistein as an anticancer agent in various types of cancer including breast cancer^4,5^, ovarian cancer^6^, and colorectal cancer^7^. It’s also worth mentioning that its safety profile is generally favorable when consumed as part of a balanced diet^8^ making it a very good candidate for being utilized as a food-additive.

Alongside all of its benefits, the aspect about which our study is interested most in genistein is attributed to its aforementioned effects of increasing vasopressin content while consumed orally^2^. Making it a perfect candidate for modeling our novel food-additive to be employed in formulating a functional-food product that is mainly addressed to patients with nocturnal enuresis, along with hyoscyamine (another natural extract that is employed as a second-line treatment for the same condition).

### Epidemiology of nocturnal enuresis

Nocturnal Enuresis, a condition commonly known as bedwetting, is affecting children worldwide, despite not being considered as a life-threatening condition, it poses long-term psychiatric risk^9^. It is abundant, with an approximate percentage of around 15 to 20% among children aged around five years, notably its prevalence is inversely proportional to the age in most cases, where the ratios are reported to drop down to 1–3% by adolescence in Egypt^10^. While globally, the prevalence is highly variable, rates of 1.4 to 28% were reported among children aged between 6 and 11 years, with indications of 52% prevalence in Jamaica and 4.3% prevalence in China^11,12^ as well as 14.9% prevalence in Turkey among 6 to 16 years aged children^13^.

Distinctively, some studies indicated fourfold increase in psychological complications to be reported amongst enuretic children when compared to their normal peers, where the International Children’s Continence Society (ICCS) reported 20 to 30% of affected children suffer at least one psychiatric disorder twice their normal peers’ chance of suffering any, with reports of likeliness for poor educational performance and an overall reduced quality of life^13,14^.

### Causes

It is worth mentioning that a family history of the condition is often reported with most cases, leading to a reasonable possibility of genetic predispositions^15^, an assumption that has been later verified by a study stating that, common genetic variants were proven to considerably contribute to nocturnal enuresis risk factors. Those were genetic variants possessing roles in bladder functioning, sleep, and the production of urine^16^. Alongside a range of other proposed potential causes i.e., altered diurnal antidiuretic-hormone (i.e., vasopressin) secretion, maturational delay, decreased bladder storage ability at night, sleep-arousal dysfunction, psychological distress, as well as age of parents and level of education^17,18^.

### Treatment protocols

Proposed treatment options vary according to multiple factors including the severity of symptoms and whether the case is diagnosed as monosymptomatic (MNE) or non-monosymptomatic nocturnal enuresis (NMNE). Where MNE is the incidence of nighttime enuresis devoid of any other accompanying symptoms e.g., incontinence^19^, and NMNE is involving other symptoms that are usually requiring to other physiological assessments to identify the underlying pathologies i.e., infections or neurologic disorders^20^.

Normally, when NMNE is identified, underlying concerns are the first to be approached with treatment and in most cases the accompanying nighttime bedwetting resolves without further interference.

However, this is not the main concern of our study as we are addressing the MNE cases, where the sole symptom is nighttime bedwetting. With many treatment strategies across the years including behavioral management strategies, night-urine alarm devices, pajamas/underwear devices, and bed devices^12^. Lately it has been widely agreed that desmopressin acetate, a synthetic hormonal analogue for vasopressin, is a good first-line treatment along with enuresis-alarm schedules for bladder voiding that is suggested for children above 7 years old only in case of non-pharmacologic measures were proven not successful with the case^9,12^.

In case of not proving efficiency, a combination of the hormonal therapy and anticholinergic therapy is introduced as a next step. Only in case of complete unresponsiveness to previous treatment strategies, the patient shall be referred to antidepressant treatment^9^ i.e., tricyclic antidepressants, despite having quite unclear mode of action on enuresis, this drug category has proven almost immediate response in cases, proposedly through altering bladder responsiveness to fullness^12^.

However, our interest here is in the second-line treatment strategy i.e., anticholinergic drugs, which is a drug category that targets overactivity of the bladder through affecting its muscarinic receptors. Despite possessing some risk factors while employed for geriatrics and adults, it has been stated that there were no records of serious risk when applied to children as well as rare incidence of side-effects^9^. Besides, there are RCTs-evidence stating that anticholinergics are an effective addition to the MNE treatment protocol^21^. Hence, our second candidate for the modeling process emerges, i.e., L-Hyoscyamine.

Hyoscyamine, is a tropane alkaloid where L-hyoscyamine is the levo-isomer of atropine that is predominantly obtained from *Solanaceae* family members. It has been long known for possessing pharmacological properties including anticholinergic effects aiding in treating diverse gastrointestinal disorders, respiratory conditions, and urinary complications through actively antagonizing muscarinic-3 receptors in smooth muscles, eventually leading to reduced bladder contractions, peristalsis, and cycloplegia as well as other conditions^22,23^. In addition to that, it has been reported to possess some immunomodulatory effects^24^. As a tropane alkaloid, hyoscyamine is rapidly absorbed and distributed through body until expelled with urine. Thus, it does not accumulate in the tissues and is reported to be neither possessing chronic toxicity nor genotoxicity. Besides the known side effects of anticholinergics at elevated dosages i.e., lack of sweating, increased heart rate, as well as salivary and nasal secretions dryness, hyoscyamine itself hasn’t show any further adverse effects as a result of long-term moderate usage^22^. Notably, its ability to reduce gastrointestinal tract distress may lead to better nutrient digestion and absorption eventually affecting overall metabolic health and wellbeing, in an indication to its potential further health benefits if employed with moderation or incorporated into a balanced diet.

Upon the aforementioned data, in this study we aimed to model and validate a food-additive that is mainly composed of the two molecules (genistein and l-hyoscyamine) that appear to approach the same problem through different routes. Where genistein is proven to increase the natural expression of vasopressin that is synthetically mimicked by the 1^st^ line treatment approach. And L-hyoscyamine i.e., anticholinergic tropane alkaloid which is employed as a 2^nd^ line treatment for the same condition as well as being also derived originally from a natural plant extract. Hence, taking into consideration the crucial rule of Density Functional Theory (DFT) descriptors in natural extract validating as well as testing newly proposed composites prior to in vitro and in vivo interventions^25^. We were able to analyze the electronic structure and reactivity profiles of our proposed composites to the modeled interaction possibilities between the two molecules. In order to understand and identify the most achievable poses on experimental bases. As well as the poses that are likely to possess the most predictable behavior in food matrix, since our main aim is to formulate functional food for patients with MNE. Aligning with the increasing consumer demand for natural health-conscious alternatives to synthetic ingredients.

The Selected DFT descriptors for modeled-structures screening process were addressed to provide an overall indication of stability in various food matrices as well as to predict their chemical activity i.e., total energies^26^, HOMO-LUMO energy gap, where greater values are often indicative of lower reactivity and higher kinetic stability^27^, binding energies were calculated to predict the favorability of interaction between the two parent molecules where negative values often suggest favorable ligands interaction^28^, total dipole moment (TDM) as an indication of hydrogen bonding ability of the composites since higher levels of TDM are often associated with enhanced ability of forming hydrogen bonds^29^.

Some thermochemical descriptors were selected to provide a more thorough insight into the behavior and stability of modeled composites in various food-processing circumstances i.e., cryogenic storage, heat treatments, solubility and reactivity in biosystems and food matrix. Enthalpy and free energy, were employed to monitor stability and intramolecular chemical reaction spontaneity^30^ in both, biosystems and food matrices. Zero-point energy (ZPE) was employed to provide an insight into the thermodynamic behavior of molecules at low-temperature surroundings i.e., cryogenic storage, since ZPE represents a system’s energy at absolute zero^31^. Entropy values were used to assess the extent to which the molecular system in the modeled composites is disordered, where lower entropy values are usually associated with less complex molecular structures and more predictable behaviors^32^. Tolerance to various heat-treatments during food processing was also assessed through heat-capacity values, where heat-capacity by definition is the amount of heat energy applied to a system (a system can tolerate) before its temperature rises by one unite change^33^.

Also, some valuable visualizations were employed in assessing important parameters based on the DFT calculations i.e., molecular electrostatic potential (MESP) mapping which provides a visual index for charge distribution across the structure^34^. As well as non-covalent interactions (NCIs) isosurface mapping, which is considered an essential aspect of molecules interactions with biosystems that directly impact their behavior and biological activity^35^. And since QTAIM framework has been accounted for as an approach to provide valuable insights into the mechanisms of atomic interactions and structures stability through clarifying bond paths and bond critical points (BCPs)^36^, a visualization has been obtained for all the structures to assess their predicted stability.

Recently computational methods show the potential to be applied in food science according to their ability to describe many factors helpful in this field^37,38^. So that, the present study in the same realm of exploration and valorization of natural extracts highlights the fact that the future of computational studies utilizing DFT descriptors in food science is promising. As it offers a road map for innovation in functional food formulation. It also underscores the growing intersection of food safety in regard to consumer’s health, as well as sustainability in food industry and research through saving valuable resources while offering precise tools.

### Calculation details

The molecular structures of parent molecules, genistein and L-hyoscyamine, were retrieved from the PubChem database. Both structures were exposed to a discrete geometry optimizations process in order to specify the most reluctant sites for non-covalent interactions. The process of geometrical optimization was performed using Gaussian 09 W^39^ and visualized using GaussView 6^40^ where the DFT/APFD method was employed^41^ in combination with the 6-311 + + G (d, p) basis set^42^. Incorporating diffuse functions and polarization effects of the basis set with the high accuracy level of the APF-D empirical dispersion model, was selected in regards to this model’s reported accuracy in describing vast section of the potential-energy surfaces of wide range of elements as well as small hydrocarbon dimers, besides its reported reproducibility for organic molecules’ relative conformational-energies and its comparability of accuracy levels to calculations carried out using the CCSD(T)/aug-cc-pVTZ method^41^ with relative considerable preservation of computational time if compared to it.

Post-optimization analyses for parent molecules were conducted to assess the electronic and reactivity properties of the parent molecules included Frontier molecular orbitals (FMOs), the band gap (ΔE), which is calculated as ΔE=E_−LUMO_–E-_HOMO_^43^, and molecular electrostatic potential (MESP) mapping were all calculated as part of the post-optimization analysis of the parent molecules. Multiwfn software^44^ was used to evaluate the global reactivity indices, which include vertical electron affinity (EA =E(N)−E(*N*+1)), vertical ionization potential (IP=E (N-1)–E(N)), chemical potential (µ), Mulliken electronegativity (χ) µ =-χ≅-IP+EA/2, hardness (η≅IP–EA), softness (S=1/η), electrophilicity index (ω=χ2/2η)^45^, and nucleophilicity index (ε = 1/ ω)^46^. Fukui functions were used to assess nucleophilic, electrophilic, and radical reactivity, represented as f_k_^+^, f_k_^−^, and f_k_°, respectively. These indices were computed as f_k_^+^ = q^k^ (*N* + 1) – q^k^(N) for nucleophilic attack, f_k_^−^ = q^k^(N) – q^k^ (*N* − 1) for electrophilic attack, and f_k_⁰ = q^k^(*N* + 1) – q_k_(*N* − 1) for radical reactivity, where q^k^ represents the electron population at the k^th^ atom in the neutral (N), anionic (*N* + 1), or cationic (N-1) state^45^. In this study, Hirshfeld charges were employed to calculate condensed Fukui functions^47^. To gain deeper insights into reactivity, the condensed dual descriptor (CDD) was employed to differentiate between electrophilic and nucleophilic attack sites. The CDD is determined using the formula: CDD = f ^+^ − f^−^^48^.

Guided by the post-optimization analysis, a literature-screening process was carried out to determine the intersections between recorded bonding-sites of L-hyoscyamine (the less reactive parent molecule). Based on the crystallography of hyoscyamine sulphate^49^, and our DFT data analysis results, the most significant binding sites that aligned were at the (C = O) on C13 and at the (-OH) at C16. As for genistein, due to the controversial effects of its high binding affinity to various estradiol receptors^50^, the crystallographic structure of genistein complexed with Erα was obtained from the protein data bank (1 × 7R). Binding sites of genistein with the receptor were compared to the results of our DFT studies. Retrieved data (from both our results and screened literature) intersected at the two active sites, hydroxyl groups (-OH) at C15 and C20. Thus, these were the sites submitted for Gen-Hyo composite modeling process. On the other hand, natural geometry of the optimized parent-structures was one critical aspect to be considered while modeling the composite. Hence, PyRx software^51^ was employed to obtain the structure with the best possible affinity via ligand-ligand docking. That produced a possible conformation of binding Affinity= -3 kcal/mol. Hence, guided by the obtained spatial pose, interactions between the two parent molecules were carried out between the C12 in genistein and the (-OH) at C16 as well as the (C = O) on C13 of hyoscyamine. Detailed summary of the modeling process is shown in (Table 1) where the interaction sites, bonds, pre and post optimization bond lengths are identified.

**Table 1:** Details of modeled composite structures where each represented a potential non-covalent interaction.

Pose	Interaction-site		Bond	Bond-lengths	
	Genistein	L-Hyoscyamine		Pre-optimization (A°)	Post-optimization (A°)
01	C20–O5–H30	C13 = O2	H30…O2	1.43797	1.77254
02	C15–O4–H29	C13 = O2	H29…O2	1.49500	1.68825
03	C12↔H21	C16-O3H41	C12…O3	2.23484	3.15105
04	C20–O5–H30	C16-O3H41	H30…O3	0.83703	1.72131
05	C15–O4–H29	C16-O3H41	H29…O3	0.92281	1.66516
06	C12↔H21	C13 = O2	C12…O2	1.06519	3.23151

– denotes covalent interaction, ↔ denotes resonance interaction, … denotes weak interaction.

Given the high computational cost of optimizing large composites at the APFD/DFT level, a more efficient approach was adopted. The composites were optimized using the DFT/B3LYP method with the 6-31G basis set^52^, striking a balance between accuracy and computational efficiency, in alignment with our composites screening and comparing aim, we targeted the most minimal details in the basis set selection without any representation of extra polarization functions, so as to investigate the extent to which the studied possible interactions are clear. The selected basis set is compatible with all the constituents of our composites^53^ where the split-valence set of 6-31G without any additional polarization functions was found to be eligible while meeting our requirement of time efficiency^54^. Thus, to maintain consistency in binding energy calculations, the parent compounds were re-optimized at the same theoretical level (DFT/B3LYP, 6-31G).

Post-optimization analysis for the modeled structures were aiming to further understand the influence of interactions on the nature of the composites as well as to predict their behavior as proposed food additives for functional-food formulation. Through assessing each of the following parameters:

### DFT descriptors

Selected DFT descriptors such as total energy, HOMO-LUMO gap i.e., (E_gap_​=E_LUMO_​−E_HOMO_​)^43^, binding energy i.e., (BE = E_complex​_−∑E_fragments_)^55^, dipole moment, polarizability were employed to provide an overall insight into the energies of modeled composites in regard to their reactivity, kinetic stability, spontaneity of interaction.

Some visual parameters based on DFT descriptors were also obtained to help further refine the screening process of modeled composites i.e., intramolecular non-covalent interactions, bond critical points in the topological surface of each composite via Multiwfn^44^. The VMD software^56^ was used to visualize the NCI-isosurfaces of the composites as well as their BCPs and bond paths. Also, the MESPs of the modeled structures were mapped and visualized using GaussView 6.0^40^ to provide an insight into the charge distribution.

### Thermodynamic parameters

Some selected thermodynamic parameters i.e., zero-point energy, enthalpy, free energy, heat capacity, entropy were assessed via gaussian09 software^39^; in order to foresee the composites’ extent of stability during various food processing and storage circumstances that are temperature-dependent, further aiding in the screening of the modeled structures and choosing the best fit.

### ADME evaluation

In alignment with the aim of modeling our candidate molecules as a food additive for functional food-formulation, theoretical ADME calculations were used to provide a more thorough insight, using SwissADME online tool^57^ key properties were calculated and compared to the corresponding parameters of parent molecules. Assessed parameters were topological polar surface area (TPSA), lipophilicity (LogP), water solubility, gastrointestinal (GI) absorption, P-glycoprotein (Pgp) substrate, and bioavailability score as well as synthetic accessibility.

The computational tools and parameters were carefully selected to align with the study’s objective of modeling stable Genistein–Hyoscyamine composites suitable for functional food applications. Geometry optimization and electronic property analysis of the parent molecules were conducted using Gaussian 09 at the DFT/APFD level with the 6-311 + + G (d, p) basis set, ensuring accurate depiction of non-covalent interactions and electronic characteristics. Both parent compounds were re-optimized, and their composites were optimized using the DFT/B3LYP method with the 6-31G basis set. This level of theory was chosen to strike a balance between computational efficiency and accuracy, particularly given the increased size and complexity of the composite structures, which would make higher-level methods computationally prohibitive. PyRx was employed for practical ligand–ligand docking to generate plausible interaction poses. Comprehensive analyses of molecular reactivity and non-covalent interactions were carried out using Multiwfn, GaussView, and VMD. Furthermore, SwissADME was utilized to assess key pharmacokinetic and physicochemical properties, especially ingestion suitability, in line with the study’s focus on developing safe and effective food additive candidates.

## Results and discussions

### Analysis of parent molecules

#### Geometry optimization

**Fig. 1:**
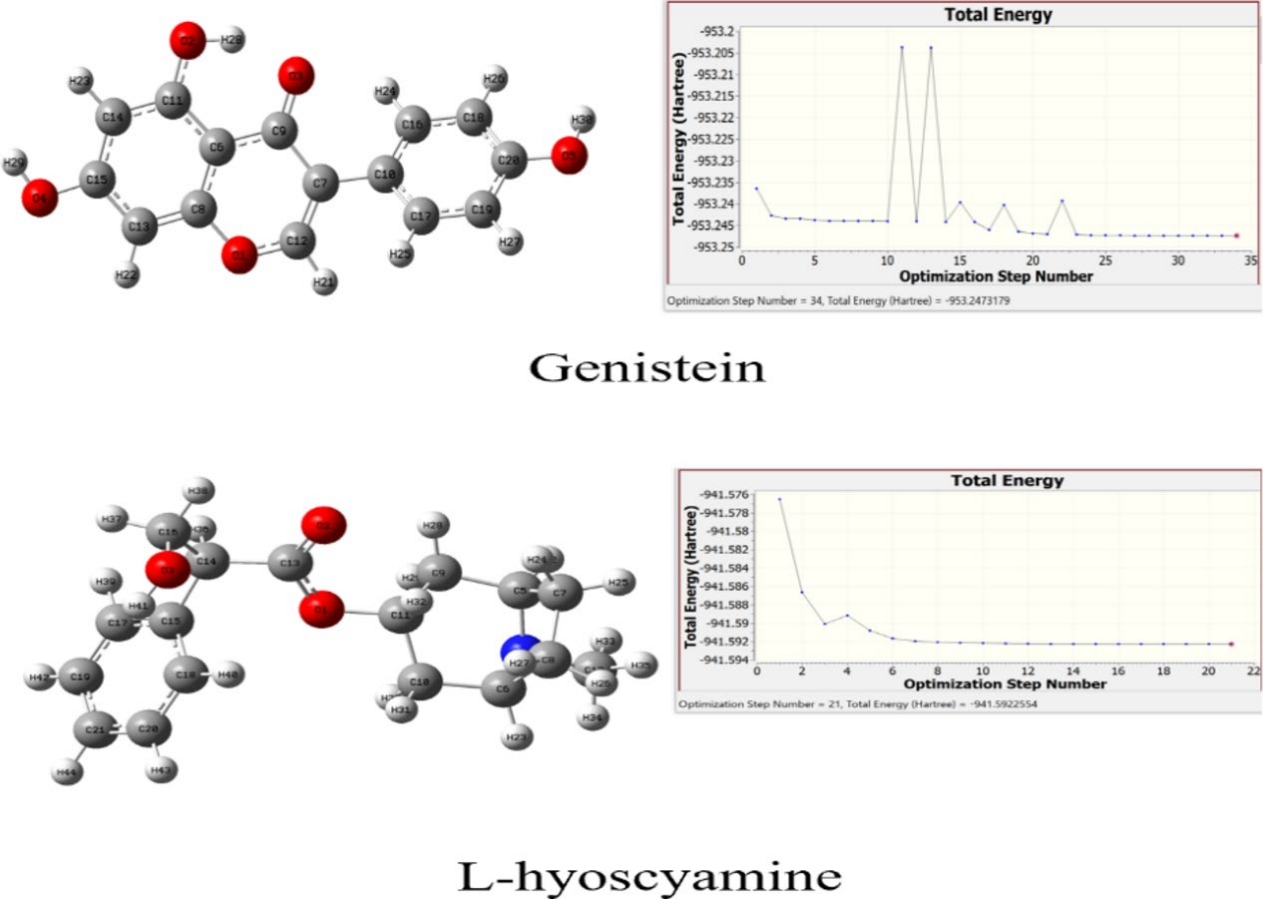
Optimization curve of Genistein and L-hyoscyamine at DFT/APFD with 6-311G++ (d, p) basis set.

Optimized structures of Genistein and L-hyoscyamine confirmed their structural stability and provided valuable insights into their conformational preferences^46,58^. The optimization curves for Genistein and L-hyoscyamine are presented in (Fig. 1), respectively.

### Molecular electrostatic potential (MESP)

**Fig. 2:**
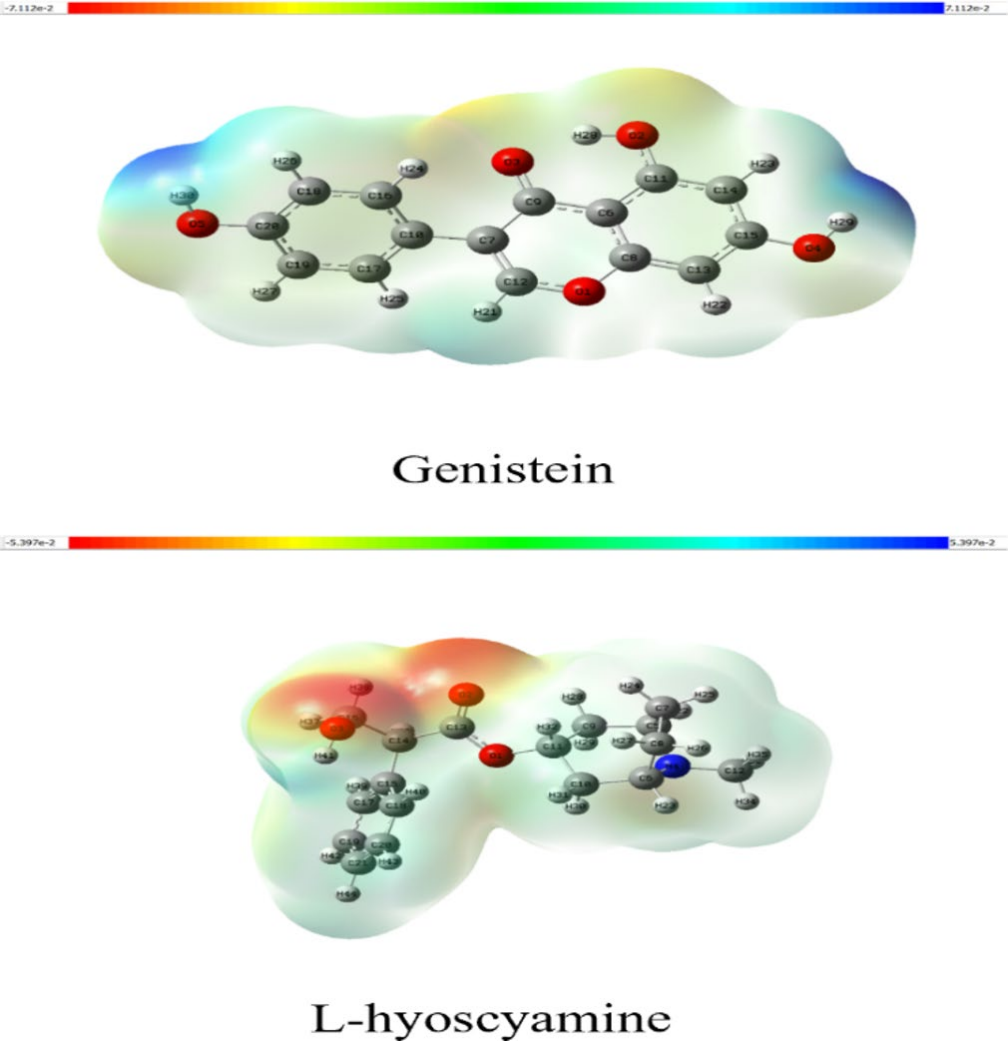
MESP of Genistein L-hyoscyamine and after optimization at DFT/APFD with 6-311G++ (d, p) basis set: red regions indicate negative potential while blue regions indicate positive potential.

The molecular electrostatic potential (MESP) map serves as a valuable tool for visualizing charge distribution and identifying potential reactive sites. The MESP map of Genistein (Fig. 2) highlights distinct regions of electrostatic potential, ranging from negative (red) to positive (blue). The blue regions, representing positive potential, are associated with the hydroxyl groups (-OH) at C15 and C20, indicating these as favorable hydrogen bond donor sites. Similarly, the MESP map of L-hyoscyamine (Fig. 2) displays regions of varying electrostatic potential. The red regions, indicative of negative potential, are localized around the carbonyl (C = O) group, identifying it as a favorable hydrogen bond acceptor site. These findings are essential for understanding the molecular interactions and predicting binding affinities with other molecules^34^.

### Frontier molecular orbital (FMO) analysis

**Fig. 3:**
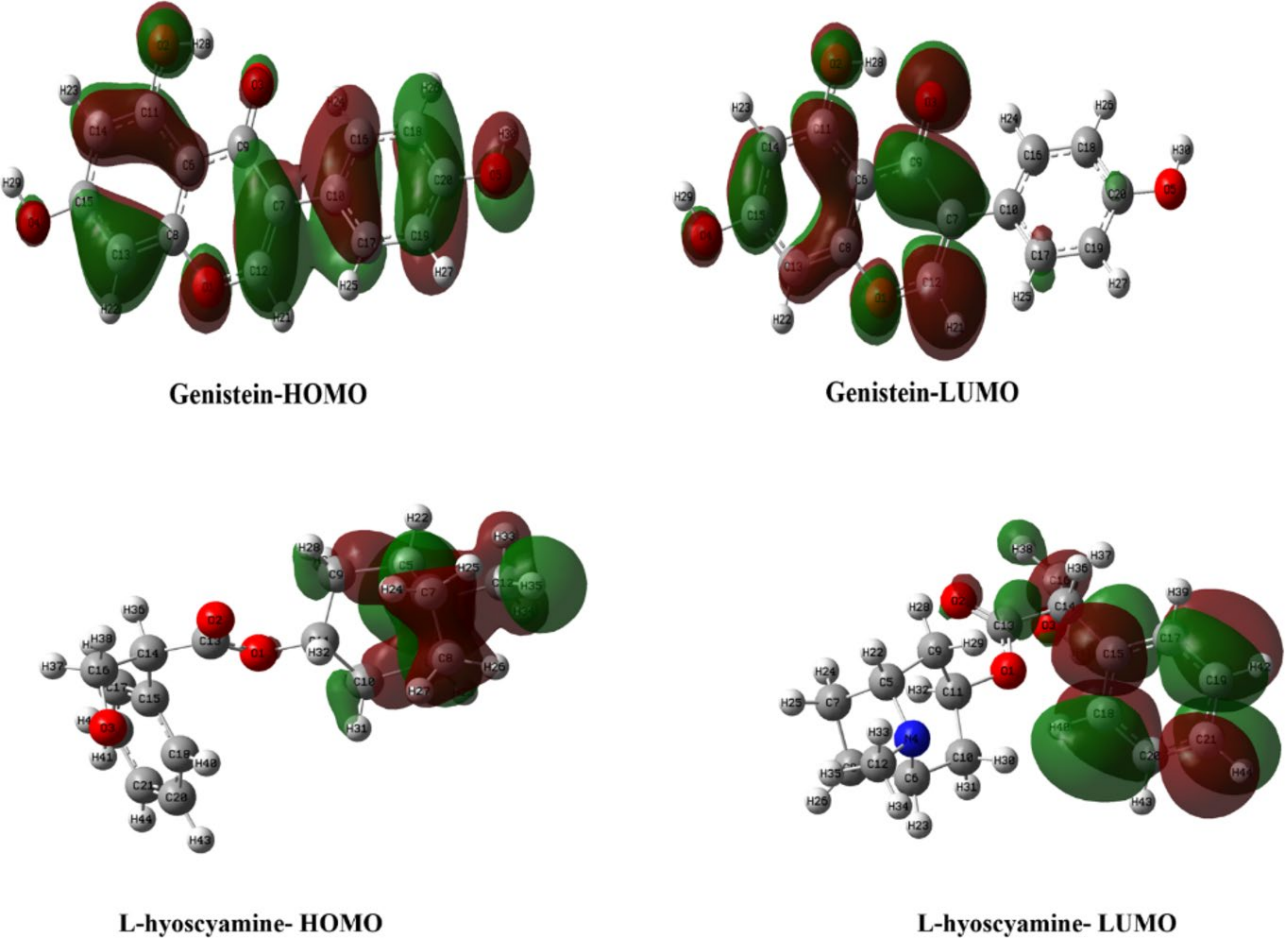
HOMO and LUMO orbitals of genistein and L-hyoscyamine after optimization at DFT/ APFD with 6-311G++ (d, p) basis set.

Frontier molecular orbitals, namely the HOMO (highest occupied molecular orbital) and LUMO (lowest unoccupied molecular orbital), provide valuable insights into chemical reactivity. The HOMO, being electron-rich, is prone to electrophilic attacks, while the LUMO, as an electron acceptor, is sensitive to nucleophilic attacks. Figure 3 illustrates the LUMOs of genistein, which are predominantly distributed over the 5,7-dihydroxy-4 H-chromen-4-one moiety, suggesting that nucleophilic attacks are likely in this region. The HOMOs, on the other hand, are scattered throughout the molecule, making it vulnerable to electrophilic interactions. For L-hyoscyamine, Fig. 3 shows that the LUMOs are localized over the 3-hydroxy-2-phenylpropanal group, indicating that nucleophilic attacks are favorable in this area. The HOMOs are distributed over the 8-methyl-8-azabicyclo [3.2.1] octane system, marking it as a favorable site for electrophilic interactions^34^.

### Global reactivity indices

Genistein and L-hyoscyamine were subjected to calculations of the global reactivity indices, which include the electrophilicity index (ω), nucleophilicity index (ε), softness (S), hardness (η), chemical potential (µ), Mulliken electronegativity (χ), vertical electron affinity (EA), and vertical ionization potential (IP). Higher reactivity index values indicate high reactivity with other molecules, making these characteristics crucial. Although Koopman’s theorem is frequently applied to obtain IP and EA from frontier molecular orbitals, orbital relaxation and electron correlation effects are not taken into consideration. Using the neutral (E(N)), anionic (E(*N* + 1)), and cationic (E(*N* − 1)) molecular energies, the electron affinity (EA) and vertical ionization potential (IP) were accurately calculated. These data were used to create global reactivity indices. The high global reactivity index values (Table 2) indicate that Genistein and L-hyoscyamine have a high level of reactivity. The global reactivity indices provide insight into the electronic properties of genistein and L-hyoscyamine. The energy values (E(N), E(*N* + 1), and E(N-1)) indicate that both molecules exhibit similar stability, with L-hyoscyamine showing a slightly lower energy for the anionic state. The HOMO-LUMO gap is larger for L-hyoscyamine (5.69 eV) compared to genistein (4.42 eV), suggesting that L-hyoscyamine is more chemically stable but less reactive. The vertical ionization potential (IP) and electron affinity (EA) values support this, with genistein having a higher EA (0.35 eV) compared to L-hyoscyamine (−0.35 eV), indicating its greater tendency to accept electrons. Mulliken electronegativity and chemical potential values suggest that genistein is slightly more electron-attracting than L-hyoscyamine. Additionally, the hardness (η) of L-hyoscyamine (8.28 eV) is higher than that of genistein (7.28 eV), reinforcing its lower reactivity, while genistein has higher softness (0.14 eV) than L-hyoscyamine (0.12 eV), indicating greater flexibility in electronic interactions. The electrophilicity index is higher for genistein (1.09 eV), making it a better electrophile, whereas L-hyoscyamine has a higher nucleophilicity index (1.16 eV), suggesting its stronger nucleophilic behavior. Overall, these findings indicate that genistein is more electrophilic and reactive, whereas L-hyoscyamine exhibits greater chemical stability with enhanced nucleophilicity.

**Table 2:** Global reactivity indices genistein and L-hyoscyamine.

Indices	Genistein	L-hyoscyamine
E(N), HA	−953	−942
E(*N* + 1), HA	−953	−942
E(*N*−1), HA	−953	−941
E_HOMO(N), (eV)	−6.24	−6.26
E_LUMO(N), (eV)	−1.82	−0.57
Band gap, (eV)	4.42	5.69
Vertical IP, (eV)	7.62	7.93
Vertical EA, (eV)	0.35	−0.35
Mulliken electronegativity, (eV)	3.98	3.79
Chemical potential, (eV)	−3.98	−3.79
Hardness, (eV)	7.28	8.28
Softness, (eV)^1−^	0.14	0.12
Electrophilicity index, (eV)	1.09	0.86
Nucleophilicity index, (eV)	0.92	1.16

### Local reactivity and Fukui function analysis

**Fig. 4:**
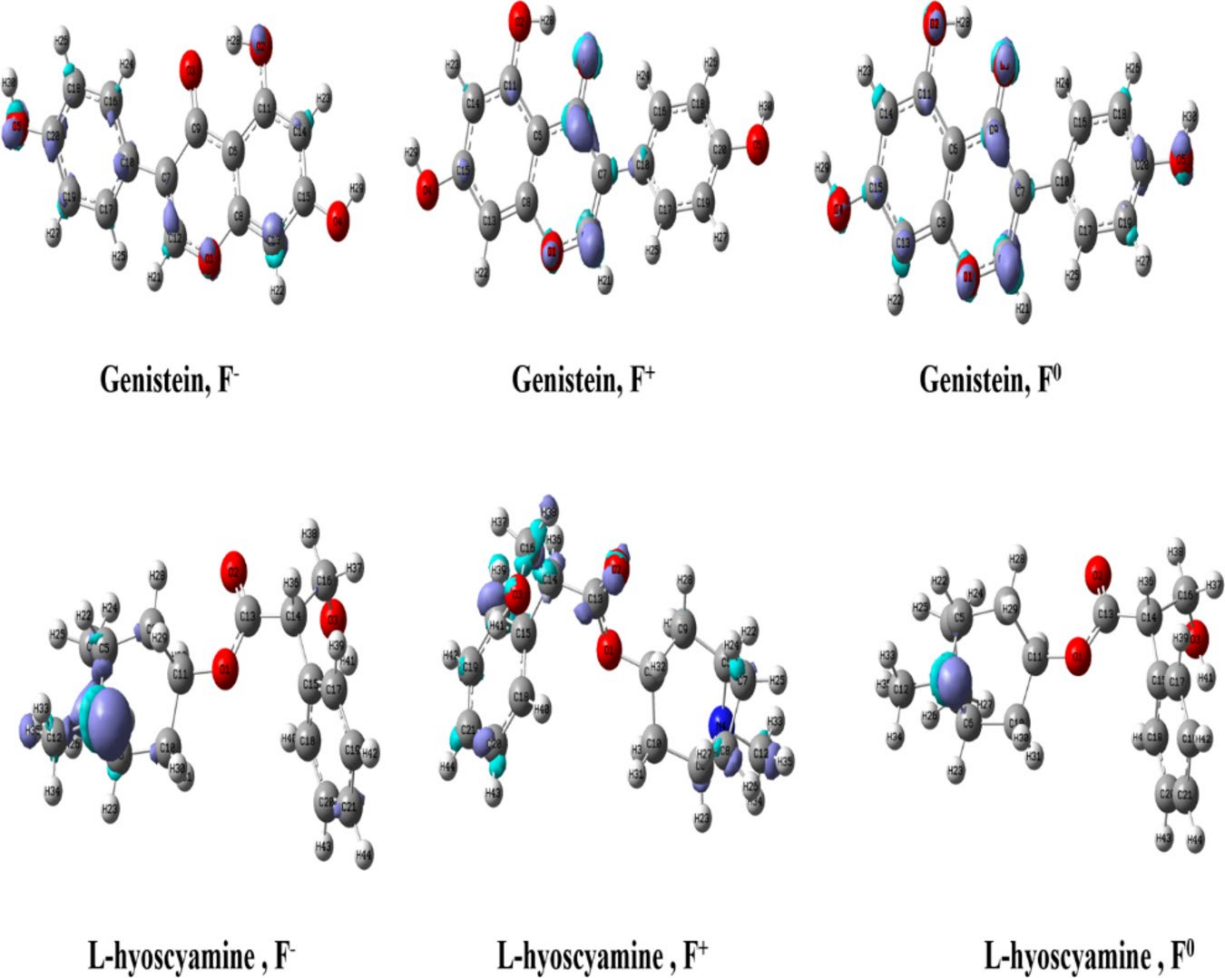
Fukui functions of Genistein and L-hyoscyamine.

The Fukui function act as local reactivity descriptors, derived from electron density while maintaining nuclear geometry unchanged. They help assess the activity and selectivity of compounds by predicting the probability of electron donation or acceptance at particular atomic sites. These functions allow for the identification of areas prone to radical, electrophilic, or nucleophilic attacks for Genistein and L-hyoscyamine (Fig. 4). A positive condensed dual descriptors (CDD) value indicates a region more prone to nucleophilic attack, whereas a negative value signifies a higher susceptibility to electrophilic attack^48^. Based on the computed CDD values for Genistein (Table 3), positive CDD values at O-3 (0.076), C-9 (0.084), and C-12 (0.071) indicate a higher tendency for nucleophilic attack. Conversely, negative CDD values at C-10 (-0.057), C-16 (-0.028), and C-20 (-0.028) suggest that these sites are more susceptible to electrophilic attacks. The balance between positive and negative CDD values highlights Genistein’s dual reactivity, making it capable of both nucleophilic and electrophilic interactions. For L-hyoscyamine (Table 4), negative CDD values at N-4 (-0.173) and C-21 (-0.028) indicate that these sites are significantly more prone to electrophilic attack. In contrast, positive CDD values at H-39 (0.062) and H-42 (0.059) suggest preferential nucleophilic reactivity at these positions. The predominance of negative CDD values implies that L-hyoscyamine is generally more susceptible to electrophilic attacks than nucleophilic reactions.

Based on the calculated global and local reactivity descriptors, genistein can be classified as an electrophile, while L-hyoscyamine acts as a nucleophile. Genistein exhibits a lower HOMO-LUMO gap (4.42 eV), higher electrophilicity index (1.09 eV), and positive electron affinity (0.35 eV), indicating a higher tendency to accept electrons. It also shows higher softness, which correlates with greater chemical reactivity. These features, combined with the presence of electrophilic reactive sites identified through the Fukui function and positive condensed dual descriptor (CDD) values at atoms like O-3, C-9, and C-12, confirm its electrophilic nature. In contrast, L-hyoscyamine demonstrates a higher nucleophilicity index (1.16 eV), higher chemical hardness (8.28 eV), and a negative electron affinity (-0.35 eV), all of which are indicative of a compound that resists accepting electrons and favors donating them. Additionally, its local reactivity analysis shows negative CDD values at key atoms such as N-4 and C-21, suggesting susceptibility to electrophilic attack and confirming its nucleophilic character.

**Table 3:** Genistein Fukui functions (local reactivity indices).

Atom	Fukui functions of genistein						
	q(*N*)	q(*N* + 1)	q(*N*−1)	f−	f+	f0	CDD
1(O)	−0.066	−0.115	−0.024	0.042	0.050	0.046	0.008
2(O)	−0.182	−0.227	−0.127	0.055	0.045	0.050	−0.010
3(O)	**−0.237**	**−0.339**	**−0.211**	**0.026**	**0.102**	**0.064**	**0.076**
4(O)	−0.170	−0.207	−0.137	0.033	0.037	0.035	0.005
5(O)	−0.183	−0.208	−0.114	0.068	0.025	0.047	−0.043
6(C)	−0.064	−0.073	−0.050	0.013	0.009	0.011	−0.004
7(C)	−0.033	−0.078	0.001	0.034	0.045	0.039	0.011
8(C)	0.082	0.068	0.095	0.013	0.015	0.014	0.002
9(C)	**0.119**	**0.025**	**0.128**	**0.009**	**0.093**	**0.051**	**0.084**
10(C)	**−0.018**	**−0.006**	**0.028**	**0.046**	**−0.011**	**0.017**	**−0.057**
11(C)	0.094	0.054	0.127	0.032	0.041	0.036	0.009
12(C)	**0.064**	**−0.052**	**0.109**	**0.045**	**0.116**	**0.081**	**0.071**
13(C)	−0.091	−0.127	−0.021	0.071	0.035	0.053	−0.036
14(C)	−0.095	−0.131	−0.054	0.042	0.036	0.039	−0.006
15(C)	0.086	0.040	0.114	0.028	0.046	0.037	0.017
16(C)	**−0.033**	**−0.040**	**0.002**	**0.035**	**0.007**	**0.021**	**−0.028**
17(C)	−0.040	−0.051	−0.010	0.031	0.010	0.020	−0.021
18(C)	−0.068	−0.088	−0.028	0.040	0.020	0.030	−0.019
19(C)	−0.060	−0.082	−0.014	0.046	0.022	0.034	−0.024
20(C)	**0.071**	**0.043**	**0.127**	**0.056**	**0.028**	**0.042**	**−0.028**
21(H)	0.064	0.006	0.087	0.022	0.058	0.040	0.036
22(H)	0.057	0.029	0.086	0.029	0.027	0.028	−0.002
23(H)	0.049	0.021	0.075	0.025	0.029	0.027	0.004
24(H)	0.045	0.039	0.065	0.020	0.006	0.013	−0.014
25(H)	0.042	0.030	0.063	0.021	0.012	0.016	−0.009
26(H)	0.044	0.025	0.070	0.027	0.019	0.023	−0.008
27(H)	0.051	0.032	0.079	0.028	0.019	0.024	−0.009
28(H)	0.121	0.102	0.136	0.015	0.019	0.017	0.004
29(H)	0.178	0.156	0.198	0.020	0.022	0.021	0.003
30(H)	0.173	0.156	0.201	0.029	0.017	0.023	−0.012

Electrophilic (F^−^), nucleophilic (F^+^), and radical attacks (F^0^). f^−^ = _qk_(N)−_qk_(*N* − 1); f^+^ = _qk_(*N* + 1)−_qk_(N); and f^0^ = _qk_(*N* + 1)−_qk_(*N* − 1). qk represents the electron population at the k^th^ atom in a neutral (N), anionic (*N* + 1), or cationic (*N* − 1) species.

Significant values are given in bold.

**Table 4:** L-hyoscyamine Fukui functions (local reactivity indices).

Atom	Fukui functions of L-hyoscyamine						
	q(*N*)	q(*N* + 1)	q(*N*−1)	f−	f+	F0	CDD
1(O)	−0.099	−0.099	−0.095	0.005	0.000	0.002	−0.005
2(O)	−0.258	−0.271	−0.228	0.030	0.013	0.022	−0.017
3(O)	−0.227	−0.236	−0.207	0.020	0.009	0.015	−0.011
4(N)	**−0.104**	**−0.106**	**0.070**	**0.174**	**0.001**	**0.088**	**−0.173**
5(C)	0.019	0.015	0.036	0.017	0.004	0.011	−0.013
6(C)	0.018	0.013	0.036	0.018	0.005	0.011	−0.013
7(C)	−0.064	−0.072	−0.041	0.023	0.008	0.016	−0.015
8(C)	−0.065	−0.074	−0.042	0.023	0.010	0.016	−0.014
9(C)	−0.067	−0.072	−0.057	0.011	0.004	0.007	−0.006
10(C)	−0.064	−0.069	−0.053	0.011	0.005	0.008	−0.007
11(C)	0.055	0.053	0.056	0.001	0.001	0.001	0.000
12(C)	−0.053	−0.070	−0.014	0.039	0.018	0.028	−0.021
13(C)	0.207	0.201	0.216	0.009	0.005	0.007	−0.004
14(C)	−0.029	−0.034	−0.022	0.007	0.005	0.006	−0.003
15(C)	−0.003	−0.007	0.021	0.024	0.004	0.014	−0.020
16(C)	0.017	0.009	0.029	0.012	0.008	0.010	−0.004
17(C)	−0.046	−0.062	−0.021	0.025	0.017	0.021	−0.008
18(C)	−0.044	−0.050	−0.034	0.010	0.006	0.008	−0.004
19(C)	−0.043	−0.064	−0.019	0.024	0.021	0.022	−0.003
20(C)	−0.042	−0.058	−0.019	0.023	0.016	0.019	−0.008
21(C)	**−0.045**	**−0.064**	**0.002**	**0.047**	**0.019**	**0.033**	**−0.028**
22(H)	0.037	0.011	0.059	0.022	0.026	0.024	0.004
23(H)	0.037	−0.007	0.059	0.021	0.044	0.033	0.023
24(H)	0.035	0.023	0.060	0.025	0.012	0.018	−0.013
25(H)	0.029	−0.013	0.050	0.021	0.042	0.031	0.021
26(H)	0.029	−0.026	0.050	0.021	0.055	0.038	0.034
27(H)	0.032	0.015	0.057	0.025	0.017	0.021	−0.008
28(H)	0.034	0.021	0.055	0.021	0.013	0.017	−0.009
29(H)	0.032	0.016	0.045	0.012	0.017	0.015	0.004
30(H)	0.036	0.023	0.047	0.011	0.012	0.012	0.001
31(H)	0.033	0.012	0.055	0.021	0.021	0.021	−0.001
32(H)	0.032	0.028	0.040	0.008	0.004	0.006	−0.004
33(H)	0.036	−0.002	0.067	0.032	0.037	0.035	0.006
34(H)	0.035	−0.010	0.067	0.031	0.046	0.039	0.014
35(H)	0.020	−0.026	0.074	0.054	0.047	0.050	−0.007
36(H)	0.052	0.014	0.063	0.010	0.039	0.024	0.028
37(H)	0.028	−0.002	0.042	0.014	0.030	0.022	0.016
38(H)	0.042	0.028	0.054	0.012	0.014	0.013	0.002
39(H)	**0.042**	**−0.035**	**0.057**	**0.015**	**0.077**	**0.046**	**0.062**
40(H)	0.042	0.032	0.048	0.006	0.010	0.008	0.003
41(H)	0.138	0.127	0.145	0.008	0.011	0.009	0.003
42(H)	**0.045**	**−0.032**	**0.064**	**0.018**	**0.077**	**0.048**	**0.059**
43(H)	0.046	0.011	0.061	0.016	0.035	0.025	0.019
44(H)	0.045	−0.001	0.067	0.022	0.046	0.034	0.024

Electrophilic (F−), nucleophilic (F+), and radical attacks (F0). f− = qk(N)−qk(*N* − 1); f + = qk(*N* + 1)−qk(N); and f0 = qk(*N* + 1)−qk(*N* − 1). qk represents the electron population at the kth atom in a neutral (N), anionic (*N* + 1), or cationic (*N* − 1) species.

Significant values are given in bold.

### Intermolecular noncovalent interaction (NCI) analysis

**Fig. 5:**
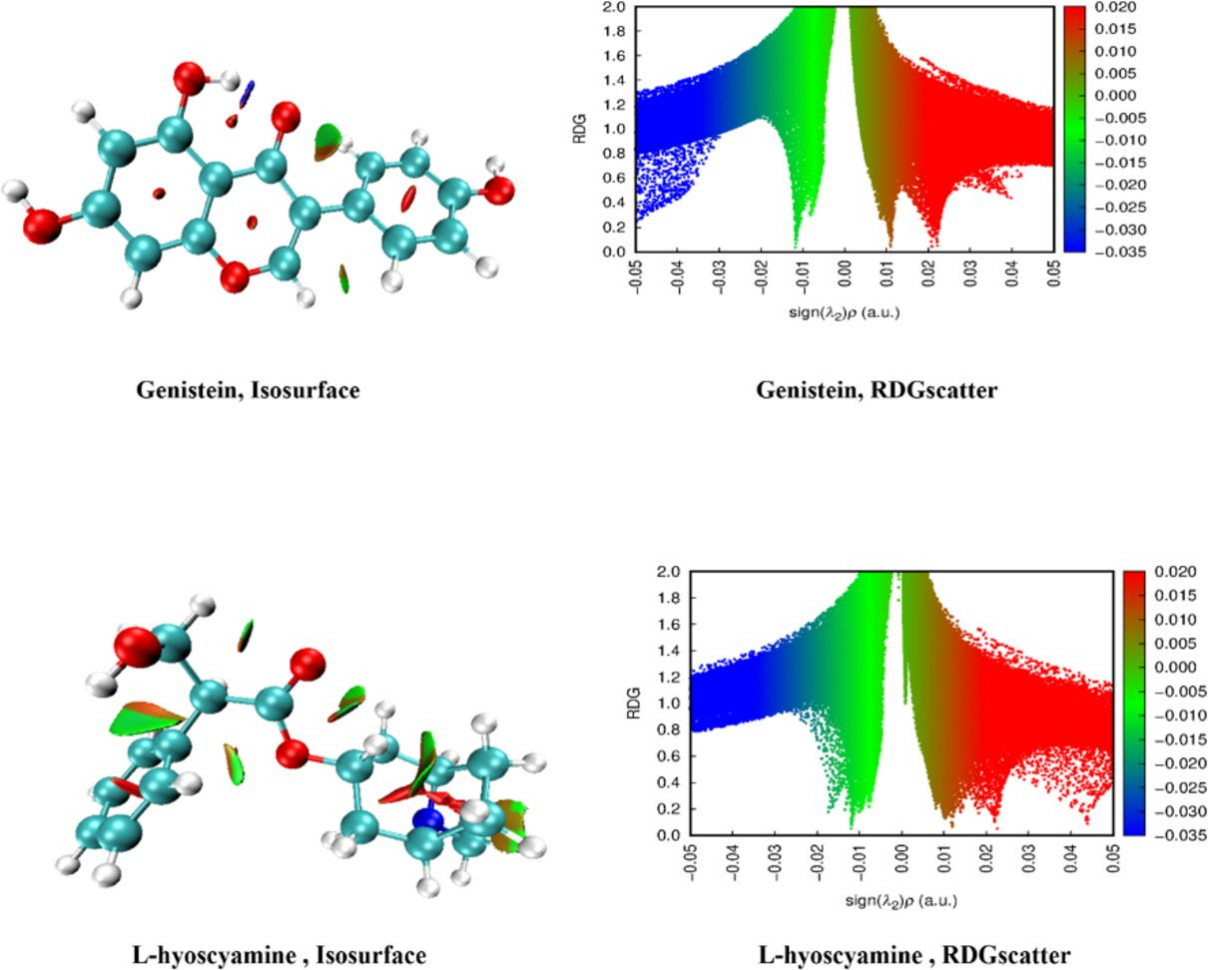
NCIs of genistein and L-hyoscyamine: positive λ indicates repulsive interactions, negative λ corresponds to attractive forces like dipole-dipole interactions and H-bonding, and λ = 0 signifies weak interactions, including Van der Waals force.

Noncovalent interactions (NCIs), including dipole-dipole forces, steric repulsion, London dispersion forces, and hydrogen bonding, are fundamental to chemical interactions between drugs and proteins, significantly impacting molecular structure and biological activity. A precise understanding of molecular geometry is crucial for recognizing and analyzing these interactions. The nature of a noncovalent interaction can be inferred from the eigenvalue (λ): a positive λ indicates repulsion, a negative λ signifies attractive interactions such as dipole-dipole forces and hydrogen bonds, while λ = 0 represents weak interactions like Van der Waals forces^35^. The analysis of Genistein based on its atomic coordinates (Fig. 5) revealed its capability to form hydrogen bonds through the hydroxyl group on C11. Additionally, it can engage in both repulsive and Van der Waals interactions, including intermolecular and intramolecular types. Similarly, the analysis of L-hyoscyamine (Fig. 5) demonstrated its ability to participate in both repulsive and Van der Waals interactions of both intermolecular and intramolecular nature.

### Energy calculations and thermochemical parameters

**Table 5:** Calculated energy values and thermochemical parameters for modeled structures.

Molecule Descriptor	01	02	03	04	05	06
Total energy (eV)	−51573.566132884	−51573.561316469	−51573.364550951	−51573.605834293	−51573.69209438	−51573.258181651
HOMO (eV)	−5.494794765	−5.616973879	−5.66731494	−5.321458249	−5.430303785	−5.498060131
LUMO (eV)	−1.700167271	−1.548055634	−1.565743034	−1.737446867	−1.579348726	−1.438121643
Band gap energy (eV)	3.794627494	**4.068918245**	**4.101571906**	3.584011382	3.850955059	**4.059938488**
Binding energy (eV)	**−0.621725694**	**−0.616909279**	−0.420143761	**−0.661427102998**	**−0.74768719**	−0.313774460999
Dipole moment (Debye)	**5.183797**	**5.998336**	4.686252	3.811547	4.843281	**5.374763**
Zero-point energy (eV)	16.503105728	16.503677167	16.479894418	16.506915322	16.513037883	16.473526954
Enthalpy (H) (eV)	17.517219586	17.515342	17.501899577	17.513437204	17.518825058	17.497845081
Free energy (eV)	14.4533266	14.483204696	14.40581552	14.524756479	14.521164577	14.411829236
Heat Capacity (Cv) (cal/mol-kelvin)	141.575	141.470	**142.205**	141.373	141.157	**142.545**
Entropy (S) (cal/mol-kelvin)	236.977	234.521	239.468	**231.163**	**231.856**	238.689

Significant values are given in bold.

As shown in (Table 5) an insight into the energies of the modeled composites was provided by calculating their total energies which notably have had a very slight variation range. The calculated energy values for HOMO-LUMO band-gap calculated at the B3LYP functional with 6-31G basis set (E_gap_=E_LUMO_−E_HOMO_)^43^ showed greater values at composites 02, 03, 06 of (4.068918245 ev), (4.101571906 ev), and (4.059938488 ev) respectively, indicating lower reactivity and higher kinetic stability which is often a desirable characteristics for our context suggesting the modeled molecule is less likely to undergo undesirable chemical reactions in the food matrix during heat processing or storage periods.

Binding energies of the modeled composites were also calculated (BE = E_complex_−∑E_fragments_)^55^ taking into consideration that the binding energies of the two parent molecules (i.e., Genistein and L-hyoscyamine) when re-optimized at the same theoretical level of (DFT/B3LYP, 6-31G) were (-25945.0554146 ev) and (-25627.88899259 ev) respectively. Despite all of the modeled structures showing negative values of binding energies suggesting the two ligands interaction is generally favorable energy wise, the fact that composites number 01, 02, 04, and 05 exhibited significantly lower binding energies of (− 0.621725694 ev), (− 0.616909279 ev), (-0.661427102998 ev), and (− 0.74768719) respectively, suggests them to be comparatively favored as they are likely to release more energy while forming their complex, leading to a more stable association. It’s also worth mentioning that in the context of food additives higher binging energies can lead to unintended chemical interactions with food matrix some of which might get affected by exposure to heat and various food-processing treatments, potentially reducing nutritional value or leading to elevated risks of hormonal disruptions^59^. Dipole moment levels of all modeled structures were also calculated showing favorable results for composites number 01, 02, and 06 equaling (5.183797 debye), (5.998336 debye), and (5.374763 debye) respectively.

The values of selected thermochemical parameters were estimated using the same computational method of DFT/B3LYP, 6-31G. As shown in (Table 5) calculated values of zero-point energies (ZPE), enthalpy, as well as free energy, they are hardly possessing any notable differences indicating narrow range of variations in stability and intramolecular chemical reaction spontaneity. Likewise, the narrow range of variations in ZPE suggests almost similar behavior of all composites in cryogenic storage circumstances. While regarding entropy values, composites number 01, 02, 04, and 05 recorded notably lower values than those of composites number 03 and 06 with 04 and 05 recording the least values. Thus, favoring composites number 04 and 05 over the rest in our context. In the meantime, the slightly higher heat capacity of composites number 03 and 06 suggests a slightly higher tolerance for food-processing heat-requiring treatments.

### ADME evaluation

**Table 6:** Calculated ADME parameters.

Molecule Descriptor	Gen	Hyo	01	02	03	04	05	06
TPSA (Å^2^)	90.9	49.77	143.83	143.83	136.76	**129.67**	**129.67**	136.76
Consensus log P (averaged of all predictions)	2.04	2.04	2.64	2.64	**2.85**	**2.75**	**3.43**	2.63
Silicos-IT class (solubility in H_2_O)	Moderately soluble	Soluble	Poorly soluble	Poorly soluble	Poorly soluble	Poorly soluble	Poorly soluble	Poorly soluble
GI absorption	High	High	Low	Low	Low	Low	Low	Low
Pgp substrate	No	No	No	No	**Yes**	No	**Yes**	**Yes**
Bioavailability score	0.55	0.55	0.55	0.55	0.55	0.55	0.55	0.55
Synthetic accessibility	2.87	4.33	6.14	6.14	**6.55**	6.14	6.14	**6.64**

Significant values are given in bold.

*Gen* genistein, *Hyo* hyoscyamine, *TPSA* topological polar surface area, *GI* gastrointestinal, *Pgp* P-glycoprotein.

As shown in (Table 6) the selected ADME parameters results are displayed. It was found that all composites had relatively low GI absorption and poor water solubility, despite that only three (i.e., 03, 05, and 06) out of six were found to be P-glycoprotein substrates. Which might lead them to be actively transported out of cells by P-glycoproteins, potentially reducing their bioavailability and efficacy through blocking permeability and causing retention, thus, completely hindering their absorption^60^. However, the other three modeled structures (i.e., 01, 02, and 04) were found to possess no ability for functioning as P-glycoproteins substrates.

Notably, the bioavailability score of all the modeled composites hasn’t changed compared to that of the parent molecules. While the synthetic accessibility of all the composites has significantly increased, with 03 and 06 recording the highest values indicating them to be the least feasible for practical application. Composites 04 and 05 recorded the least TPSA values of 129.67, suggesting that they possess the best oral bioavailability in the proposed structures as well as the most susceptibility to be absorbed in the gastrointestinal tract due to relatively higher lipid solubility compared to other composites^61^. This corresponds to the results of calculated average Log P values of composites 03, 04, and 05 of (2.85), (2.75), and (3.43) respectively, indicating them to be possessive of the highest values regarding lipophilicity.

The observed reduction in water solubility can largely be attributed to the increased molecular complexity and size of the composites resulting from non-covalent interactions between genistein and L-hyoscyamine. These interactions (particularly hydrogen bonding and van der Waals forces) stabilize key functional groups within the composite, thereby reducing the number of free hydrogen bond donors and acceptor sites available for interaction with water molecules. Furthermore, the notable rise in consensus Log P values (from approximately 2.04 for both parent compounds to as high as 3.43 in some composites) indicates a significant increase in lipophilicity, reflecting a shift in the hydrophilic-lipophilic balance. This shift suggests a preference for lipid environments, potentially improving interactions with hydrophobic regions in food matrices or biological membranes but concurrently reducing aqueous solubility. In future studies, we intend to explore formulation strategies (such as encapsulation or emulsification) to enhance water solubility and ensure practical applicability in functional food systems.

### MESP, NCI-isosurfaces, and BCPs mapping

#### MESP

**Fig. 6:**
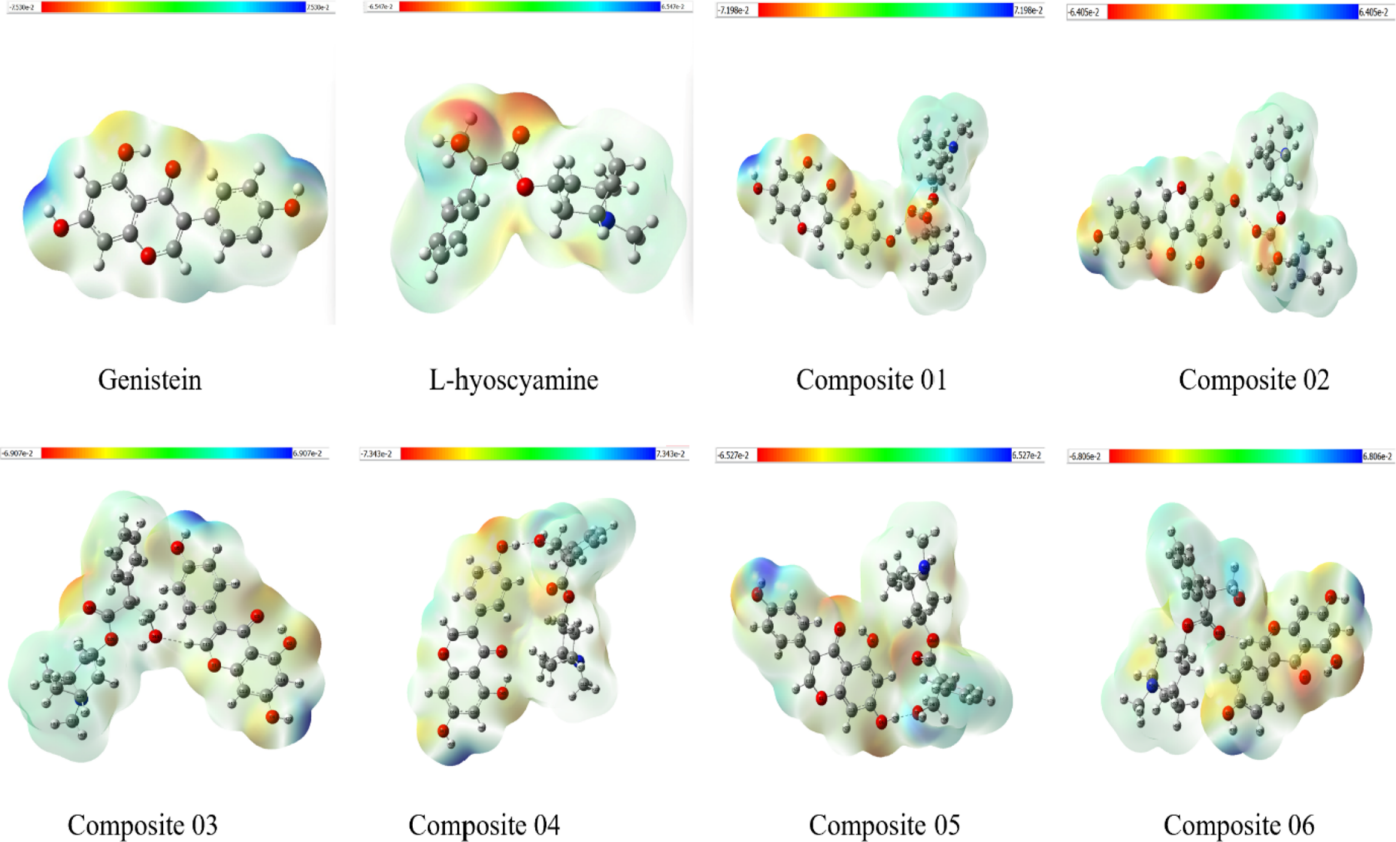
MESP of Genistein L-hyoscyamine and after reoptimization and the composites at DFT/B3LYP method with the 6-31G basis set: red regions indicate negative potential while blue regions indicate positive potential.

MESP mapping was employed to investigate the change in the potential active sites within the composites due to the different proposed interaction poses (Fig. 6). Where red is signifying negative potentials i.e., prospect electron-acceptor sites, and blue denoting the regions of positive potential in the structure where it’s more likely to act as a donor in hydrogen bonding. It is clear how the distinctively pronounced variation in the charge distribution nature of the two parent molecules has partially diminished in the majority of the composites. Thus, indicating a more even distribution across the structures. This is most obvious at composites number 02, as well as 04, 05, and 06. Despite 04, 05, and 06 exhibiting slightly more dense distribution of charges localized around previously recorded active sites of genistein side of the composite. While composites number 01 and 03 have shown more dense charges distribution localized around previously recorded active sites in both parent molecules.

#### NCIs

**Fig. 7:**
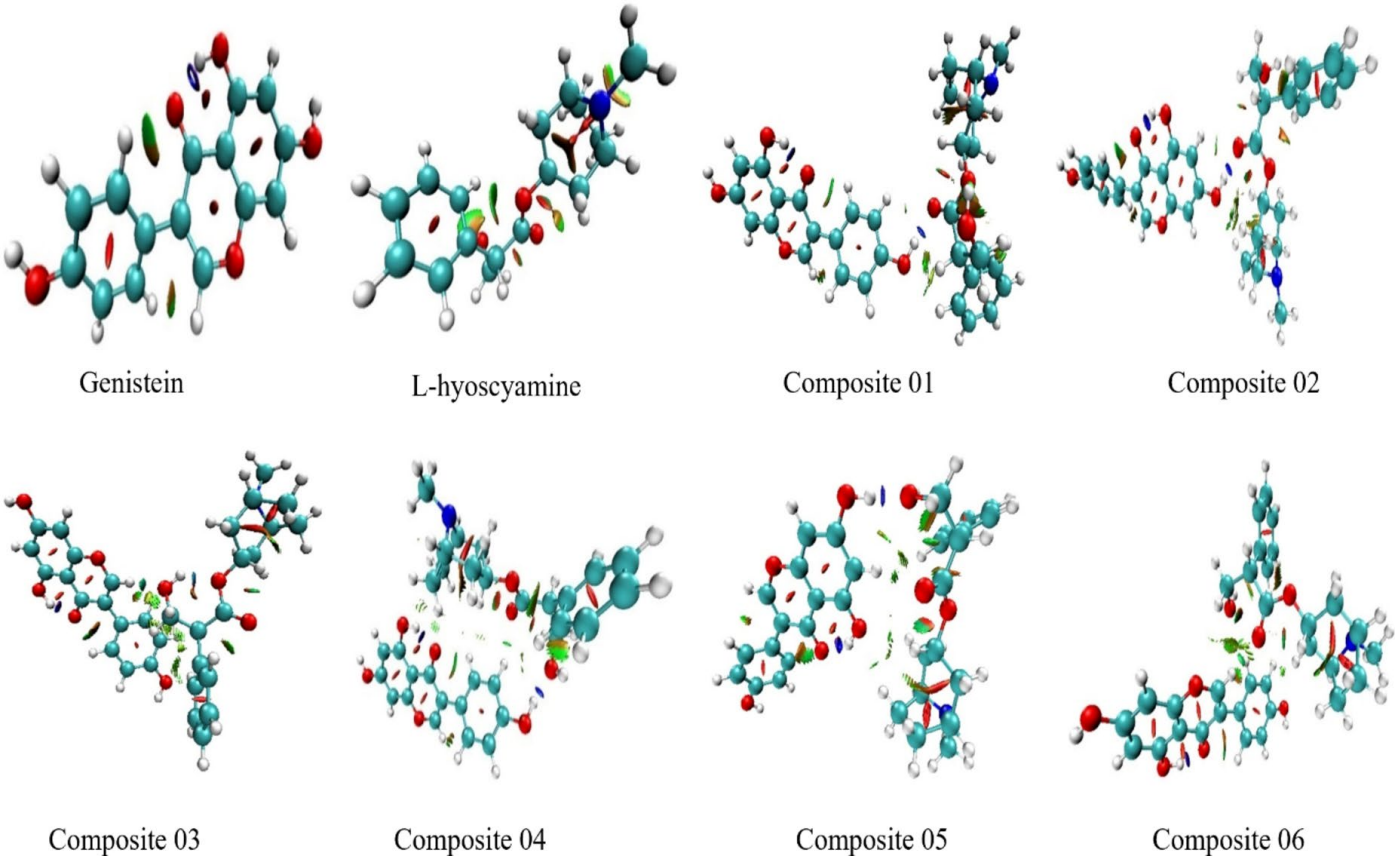
Isosurfaces for genistein L-hyoscyamine and after reoptimization and the composites at DFT/B3LYP method with the 6-31G basis set: red color indicates repulsive interactions, blue corresponds to attractive forces like dipole-dipole interactions and H-bonding, and green color signifies weak interactions, including Van der Waals force.

To further investigate the nature and strength of non-covalent interactions within the modeled composites, NCI isosurface analysis was conducted and visualized using VMD. These surfaces were color-coded, providing insight into the type and intensity of interaction: blue regions indicate strong attractive forces, such as hydrogen bonding and dipole-dipole interactions; green regions represent weak attractive interactions, primarily van der Waals forces; and red regions indicate steric repulsion. As shown in Fig. 7, composites 01, 02, 04, and 05 displayed prominent blue regions between the interacting functional groups of genistein and L-hyoscyamine, confirming the presence of strong and directional hydrogen bonds. This supports the observed low binding energy for these structures, suggesting they are more stable and better suited for functional applications. In contrast, composites 03 and 06 showed minimal or absent blue isosurfaces, with interaction regions primarily represented by green zones, indicating the dominance of weak van der Waals interactions. This observation aligns with their higher entropy values and lack of hydrogen bonding, as indicated by MESP analyses. By linking these visual features to quantitative descriptors, the NCI analysis substantiates the ranking of composite stability and reinforces the conclusion that composites 02 and 04 offer the most favorable interaction profiles for further development.

#### BCPs

**Fig. 8:**
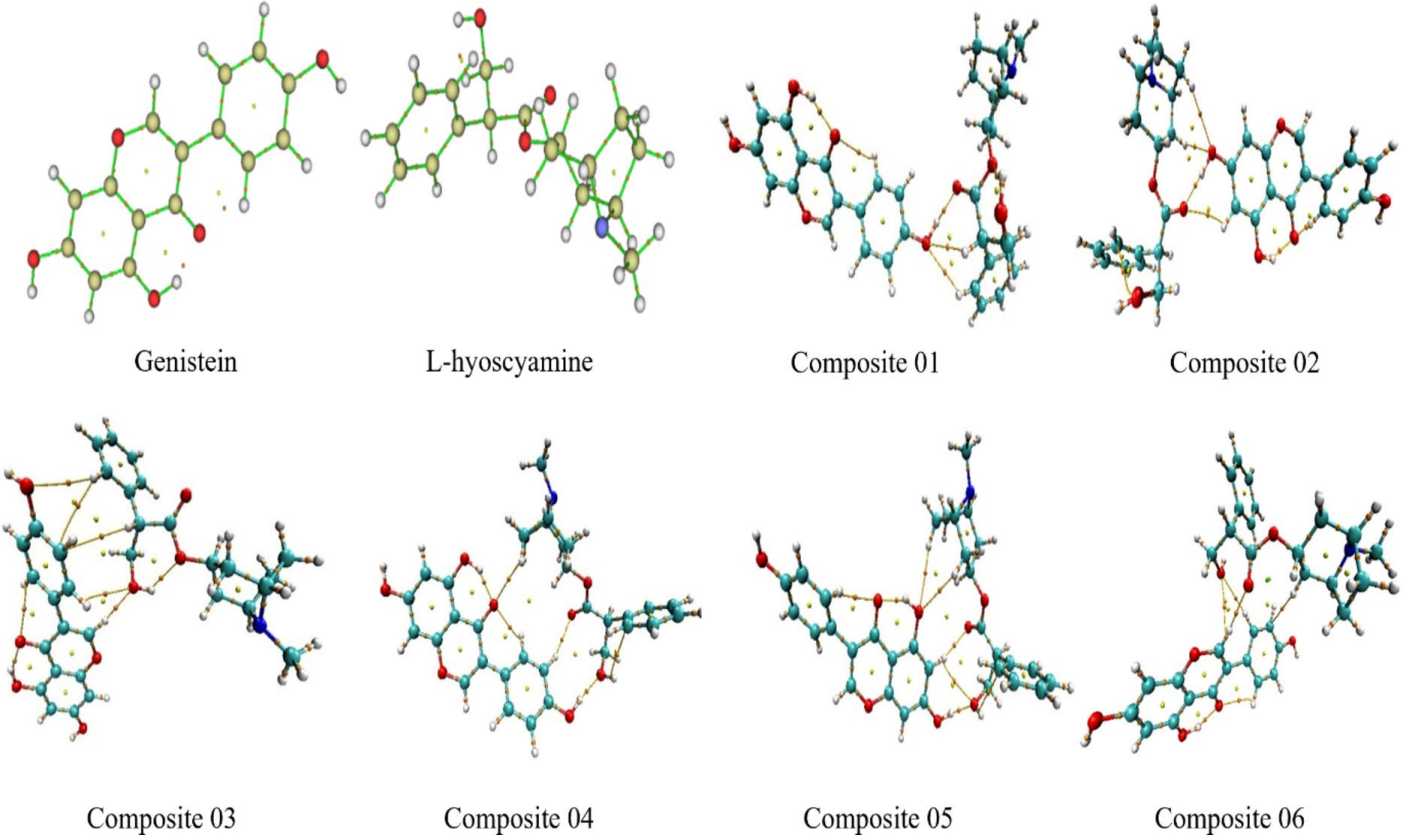
BCPs genistein L-hyoscyamine and after reoptimization and the composites at DFT/B3LYP method with the 6-31G basis set.

To further validate the intermolecular interactions and stability of the modeled composites, the Quantum Theory of Atoms in Molecules (QTAIM) was applied. This analysis provides a rigorous topological approach to visualize and confirm the presence and nature of bonding through bond critical points (BCPs) and bond paths. In this study, bond paths (yellow lines) and BCPs (orange dots) were successfully mapped using the Multiwfn package and visualized through VMD software. As illustrated in Fig. 8, all six composite structures demonstrated new intermolecular bond paths between genistein and L-hyoscyamine, confirming the formation of non-covalent interactions. The existence of BCPs in these regions implies that electron density is concentrated between the interacting atoms, substantiating the presence of stable interactions. The absence of missing BCPs further confirms that no significant bond cleavage occurred during the optimization process, underscoring the composites’ structural integrity and thermodynamic stability. Importantly, the QTAIM results align well with the observed MESP and NCI analyses. In particular, the composites that exhibited pronounced hydrogen bonding (such as composites 02 and 04) showed well-defined BCPs between donor and acceptor atoms, correlating with their favorable dipole moments, binding energies, and entropy values. This consistency strengthens the argument that these composites are both structurally stable and chemically favorable for functional food applications.

## Conclusion

In this study, we aimed to model a novel food-additive that serves formulating a functional food for nocturnal enuresis patients employing two natural extracts (Genistein and L-hyoscyamine). Through DFT calculations and ADME evaluation, composites 02 and 04 were identified as the most promising candidates for further development. Both composites demonstrated favorable ligand interactions, indicated by low binding energies, and were predicted to be non-substrates of P-glycoprotein, suggesting improved oral bioavailability. Composite 02 showed higher band gap energy, dipole moment, and heat capacity, while composite 04 exhibited superior lipophilicity, lower topological polar surface area, and reduced enthalpy, free energy, and entropy, signaling greater stability and absorption potential. Although other composites (03 and 06) showed competitive individual parameters, they were excluded due to predicted P-glycoprotein interactions, higher synthetic complexity, elevated entropy values, and lack of significant hydrogen bonding based on isosurface analysis factors that limit their suitability in food systems. These results suggest that composites 02 and 04 are promising candidates for further development.

Considering that this phase of the research aimed to screen the modeled composites for the best match in regard to the study objective, it’s imperative for the next phase to further assess the interaction between the two parent molecules in the selected composites to attain better stability in food matrices and enhance their bioavailability profiles.

As a next step, experimental validation using FT-IR and XRD is planned to confirm the modeled interactions, followed by employing the composites in food-formulations so as to prepare for in vivo studies assessing biological efficacy and safety.

Overall, this work supports the growing role of computational modeling in functional foods design with a more sustainable approach for resources exploitation. Also, it highlights the potential of natural compound-based composites to address specific health conditions through targeted dietary solutions.

## Data Availability

Primary and supplementary data supporting the results of this study will be made available upon reasonable request through Rana Abd-ElSalam via email: ranaashokr@gmail.com.
